# A Stress-Associated Protein, *PtSAP13*, From *Populus trichocarpa* Provides Tolerance to Salt Stress

**DOI:** 10.3390/ijms20225782

**Published:** 2019-11-17

**Authors:** Jianbo Li, Pei Sun, Yongxiu Xia, Guangshun Zheng, Jingshuang Sun, Huixia Jia

**Affiliations:** 1 Experimental Center of Forestry in North China, Chinese Academy of Forestry, Beijing 102300, China; yxxia@caf.ac.cn (Y.X.); guangshunzheng@caf.ac.cn (G.Z.); sjshuang@caf.ac.cn (J.S.); 2 State Key Laboratory of Tree Genetics and Breeding, Chinese Academy of Forestry, Beijing 100091, China; Sunpei@caf.ac.cn; 3 Research Institute of Forestry, Chinese Academy of Forestry, Beijing 100091, China

**Keywords:** stress-associated protein, expression analysis, transgene, salt tolerance, transcriptome, *Populus trichocarpa*

## Abstract

The growth and production of poplars are usually affected by unfavorable environmental conditions such as soil salinization. Thus, enhancing salt tolerance of poplars will promote their better adaptation to environmental stresses and improve their biomass production. Stress-associated proteins (SAPs) are a novel class of A20/AN1 zinc finger proteins that have been shown to confer plants’ tolerance to multiple abiotic stresses. However, the precise functions of *SAP* genes in poplars are still largely unknown. Here, the expression profiles of *Populus trichocarpa SAPs* in response to salt stress revealed that *PtSAP13* with two AN1 domains was up-regulated dramatically during salt treatment. The β-glucuronidase (GUS) staining showed that *PtSAP13* was accumulated dominantly in leaf and root, and the GUS signal was increased under salt condition. The *Arabidopsis* transgenic plants overexpressing *PtSAP13* exhibited higher seed germination and better growth than wild-type (WT) plants under salt stress, demonstrating that overexpression of *PtSAP13* increased salt tolerance. Higher activities of antioxidant enzymes were found in *PtSAP13*-overexpressing plants than in WT plants under salt stress. Transcriptome analysis revealed that some stress-related genes, including *Glutathione peroxidase* *8*, *NADP-malic enzyme 2*, *Response to ABA and Salt 1*, *WRKYs*, *Glutathione S-Transferase*, and *MYBs*, were induced by salt in transgenic plants. Moreover, the pathways of flavonoid biosynthesis and metabolic processes, regulation of response to stress, response to ethylene, dioxygenase activity, glucosyltransferase activity, monooxygenase activity, and oxidoreductase activity were specially enriched in transgenic plants under salt condition. Taken together, our results demonstrate that *PtSAP13* enhances salt tolerance through up-regulating the expression of stress-related genes and mediating multiple biological pathways.

## 1. Introduction

Soil salinization is a major environmental constraint on plant growth, development, and production [1]. High salinization limits the ability of plants to extract water and disrupt ion distribution, and usually causes oxidative stresses [2,3]. Worldwide, there are at least 800 million hectares of land subjected to salinity, accounting for ~6% of the land surface [3]. Due to intensive land use, lack of freshwater resources, and irrational irrigation, the salinized area is increasing. To overcome the detrimental effects of salt stress, plants have developed multiple complex strategies, including ion sequestration and/or exclusion, osmotic adjustment, metabolic adjustment, and antioxidative defense under salt stress. Several salt tolerance-related genes, such as *Salt*-*Overly*-*Sensitive* [4], *Na^+^*/*H^+^ Exchanger* [5,6], *High-affinity K^+^ transporters* [7], *NAC* [8], *WRKY* [9,10], and *AP2/ERF* [11,12], have been characterized to improve salt tolerance in some plant species. However, salt response and tolerance are complex regulatory networks involving the coordinated action of multiple genes [13]. Thus, more efficient genes need to be identified to facilitate the cultivation of varieties with high salinity tolerance.

The *stress-associated protein* (*SAP*) genes encode a novel class of zinc finger proteins and widely present in eukaryotes. Since the *OsSAP1* was firstly isolated in plants, their homologs genes have been identified in *Arabidopsis thaliana* [14], *Oryza sativa* [14], *Populus euphratica* [15], *Medicago truncatula* [16], and *Brassica napus* [17]. The essential characteristic of SAP protein is the presence of two special zinc finger domains (A20 domain and/or AN1 domain). The A20 zinc finger domain has Cx_2__-4_Cx_11_Cx_2_C consensus sequence at the N-terminus [18]. The AN1 zinc finger domain has two types, including Cx_2_Cx_9-12_Cx_1-2_Cx_4_Cx_2_Hx_5_HxC consensus sequence and Cx_4_Cx_9-12_Cx_1-2_Cx_4_Cx_2_Hx_5_HxC consensus sequence at the C-terminus [19].

To date, several *SAP* genes with one A20 domain and one AN1 domain from different species have been confirmed to be involved in tolerance to multiple abiotic stresses. In rice, the expression of *OsSAP1* is induced by different stresses (cold, drought, salt, heavy metals, ABA, and injury), and overexpression of *OsSAP1* improves the tolerance to drought, cold, and salt stresses, and enhances its immunity to pathogen in transgenic plants [20,21,22]. The transgenic plants overexpressing *OsSAP8*, *OsSAP9,* and *OsSAP11* also showed high tolerance to salt, drought, and cold stresses [23,24,25]. In *Arabidopsis*, the expression of *AtSAP5* is induced by salt, osmotic, drought, and cold stresses. Constitutively, overexpression of *AtSAP5* significantly increases tolerance to salt, drought, and heat stresses by up-regulating the expression of the stress tolerance genes [26,27]. Overexpressing *Aeluropus littoralis AlSAP* enhances the tolerance to cold, drought, and salt stresses through maintenance of photosynthesis in transgenic rice [28], and increases rice grain yield under drought condition [29]. In addition, *SbSAP14* from *Sorghum bicolor* [30] and *MusaSAP1* from banana cv. *Karibale Monthan* [31] also confer tolerance to different stresses. Except for the function investigations of SAPs containing A20 and AN1 domains, AtSAP13 with two AN1 domains and one C2H2 domain is up-regulated under ABA, salt, and Cd stresses, and *AtSAP13*-overexpressing plants exhibit high tolerance to drought, salt, and toxic metals [32]. Wheat *TaSAP17*-*D* with two AN1 domains and two C2H2 domains is induced by salt, drought, and cold stresses, and it can enhance tolerance to salt stress in transgenic *Arabidopsis* [33]. Despite these advances, these studies mainly concentrated on herbaceous plants, and the biological functions of *SAP* genes in woody plants is far from complete.

During their long life spans, perennial woody plants have evolved sophisticated systems to cope with salt stress. In view of the key roles of *SAP* genes in plant stress responses, deciphering the *SAP* functions in woody plants will be helpful for better understanding their salt response mechanisms. As a model woody plant, poplar has great economic and ecology benefits. Due to its rapid growth and high biomass yield, poplar is used widely to generate wood, fiber, feedstock for biofuels, and other bioproducts [34,35]; and it also plays important roles in soil and water conservation, sand break, carbon fixation, phytoremediation, and so on [34,35]. To our knowledge, only stress-associated protein 1 (*PagSAP1*) from *Populus alba* × *P. glandulosa* has been investigated, which showed down-regulation of *PagSAP1* increases salt stress tolerance in poplar [36]. In this study, the expression profiles of *P. trichocarpa PtSAPs* were surveyed to screen candidate genes involved in salt stress tolerance, and we found that *PtSAP13* with two AN1 domains was up-regulated dramatically under salt treatment. Thus, we generated the p*PtSAP13::GUS* and p*35S::PtSAP13* transgenic *Arabidopsis* plants to investigate the *PtSAP13* function. Moreover, RNA-seq was performed to explore the molecular mechanisms of *PtSAP13* in salt tolerance. Our research indicates that *PtSAP13* plays a positive role in salt tolerance.

## 2. Results

### 2.1. Phylogenetic Analysis of SAPs in P. trichocarpa and Other Species

**Figure 1:**
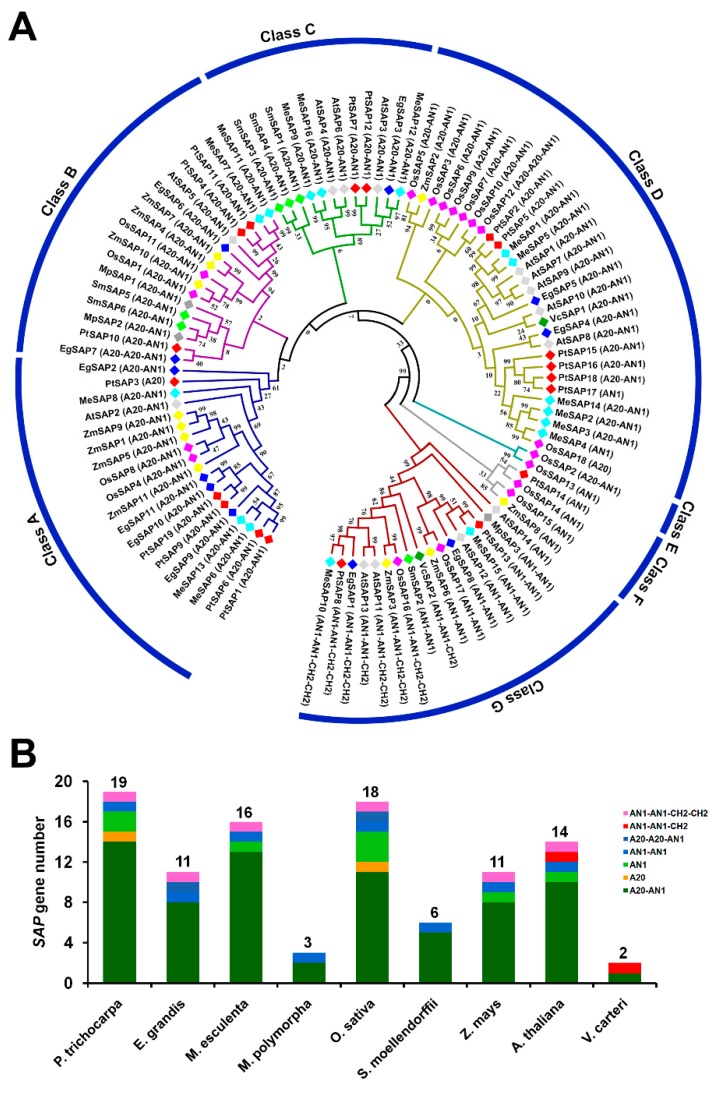
The *SAP* gene family underwent gene expansion in evolution history. (**A**) Phylogenetic tree and evolutionary relationship of the SAPs from *Populus trichocarpa* (Pt), *Eucalyptus grandis* (Eg), *Manihot esculenta* (Me), *Marchantia polymorpha* (Mp), *Oryza sativa* (Os), *Selaginella moellendorffii* (Sm), *Zea mays* (Zm), *Arabidopsis thaliana* (At), and *Volvox carteri* (Vc). (**B**) The number of *SAP* genes and zinc finger domains in different plants. The detailed *SAP* gene information and protein sequences are listed in Table S1.

A total of 19 *SAP* genes were identified from *P. trichocarpa* genome, which is in keeping with previous research [19]. Based on their locations in the genome, we sequentially named them from PtSAP1 to PtSAP19 (Table S1). Conserved domain analysis revealed that two PtSAPs (-14 and -17) included one AN1 domain (AN1); PtSAP3 contained one A20 domain (A20); PtSAP13 contained two AN1 domains (AN1–AN1); PtSAP8 contained two AN1 domain, and two CH2–CH2 domains (AN1–AN1–CH2–CH2); and the remaining 14 SAP members included one A20 domain and one AN1 domain (A20–AN1) (Table S1, Figure 1 and Figure S1). To reveal the evolutionary pattern and phylogenetic relationships of SAP, an unrooted phylogenetic tree was constructed using the protein sequences of SAPs from *P. trichocarpa* and another eight species (*Eucalyptus grandis*, *Manihot esculenta*, *Marchantia polymorpha*, *Oryza sativa*, *Selaginella moellendorffii*, *Zea mays*, *Arabidopsis thaliana*, and *Volvox carteri*) (Figure 1A). The result shows that the SAPs were classified into seven classes (Class A–Class G). The SAPs containing A20–AN1 were present in Class A–Class E, and almost all of SAPs in Class F and Class G contained AN1 and/or CH2 domains, including AN1, AN1–AN1, AN1–AN1–CH2, or AN1–AN1–CH2–CH2 (Figure 1A). Except Class E, the 19 PtSAPs were unevenly distributed in the other six classes. The SAP members of each species were counted (Figure 1B). The lower algae *V. carteri* had the minimum number of SAPs, with only two members, while *P. trichocarpa* had the maximum number of SAPs (19), followed by *O. sativa* (18), *M. esculenta* (16), *A. thaliana* (14), *E. grandis* (11), and *Z. mays* (11) (Figure 1B).

### 2.2. Expression Profiles of PtSAP Genes

Revealing the expression profiles in various tissues under stress conditions will provide clues for gene potential functions; thus, we firstly analyzed the expression patterns of *PtSAPs*. Based on the public database of *Populus*, the expression profiles of *PtSAPs* in different tissues and developmental processes, including leaf expansion, germination of bud, male/female development, and different nitrogen nutrition treatment in root and stem, were investigated (Figure S2). Three *PtSAPs* (-*2*, -*10*, and -*11*) were more highly expressed in root than other tissues, and three *PtSAPs* (-*4*, -*11*, and -*13*) were induced under ammonia, nitrate, or urea condition in root (Figure S2). Only five *PtSAPs* (-*4*, -*10*, -*11*, -*12*, and -*13*) were highly expressed in the middle of male catkin development. In the process of pre-dormant bud I to fully open bud, the expression of three *PtSAPs* (-*2*, -*5*, and -*13*) increased early and then declined, which indicates that these genes might be involved in dormant bud formation (Figure S2).

**Figure 2:**
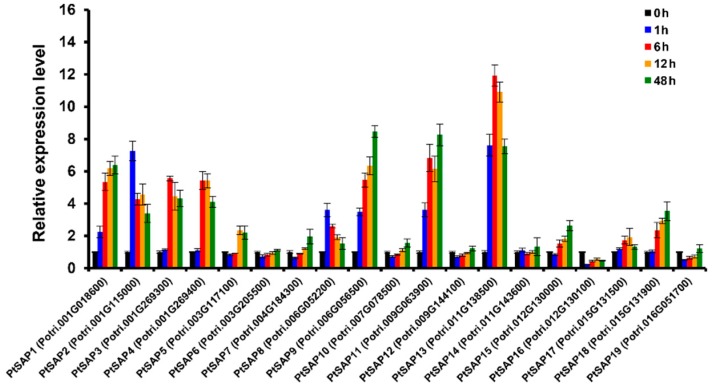
Expression analysis of *PtSAP* genes under salt stress. The gene expression levels under salt stress were determined using qRT-PCR. Error bars represent the standard deviations of three biological replicates and four technical replicates.

In addition, we detected the expression patterns of *PtSAPs* in response to salt stress using qRT-PCR. Except *PtSAP16*, the expressions of other *PtSAPs* were increased under salt treatment. Seven *PtSAPs* (-*1*, -*2*, -*3*, -*4*, -*9*, -*11*, and -*13*) exhibited significant up-regulation (Figure 2). Notably, *PtSAP13* was rapidly induced (~8-fold) at 1 h and reached its maximal level (~12-fold) at 6 h, after which, it began to decline slowly at 24 h and 48 h (Figure 2).

### 2.3. Expression Patterns of PtSAP13

**Figure 3:**
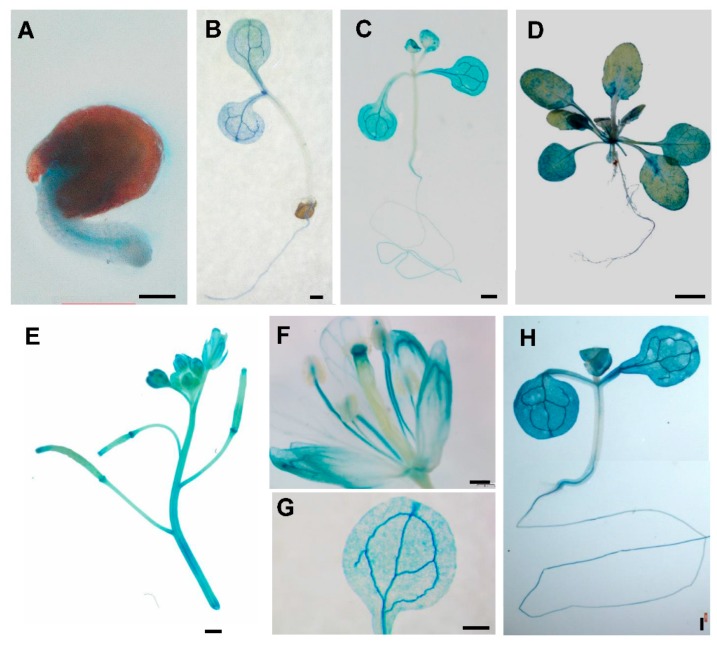
Spatiotemporal characterization of *PtSAP13* in p*PtSAP13::GUS* transgenic *Arabidopsis*. (**A**) 1-day-old seedling; (**B**) 3-day-old seedling; (**C**) 10-day-old seedling; (**D**) 1-month-old seedling; (**E**,**F**) flower; and (**G**) leaf. (**H**) The GUS staining of a 10-day-old seedling of p*PtSAP13::GUS* was treated with salt. Scale bars: (**A**) 500 μm; (**B**–**D**) 1 mm; (**E**) 5 mm; (**F**) 1 mm; (**G**) 250 μm; and (**H**) 1 mm.

According to the expression level of *PtSAP13* under salt treatment, we chose *PtSAP13* for further function analysis. The full length CDS of *PtSAP13* was 450 bp and it encoded a 149 amino acid protein with two AN1 domains (Table S1). The molecular weight of PtSAP13 was 16.97 kDa and its predicted isoelectric point was 9.02, respectively (Table S1). In order to further determine the expression patterns of *PtSAP13*, the promoter fragment was amplified and constructed into the pMDC164 vector to fuse with the reporter gene GUS (namely, p*PtSAP13*::*GUS*), and transgenic *Arabidopsis* was generated. GUS staining in *Arabidopsis* revealed that GUS signal was detected in roots in 1-day-old seedlings (Figure 3A). Subsequently, 3-day-old seedlings showed high GUS signal in cotyledons and roots (Figure 3B), as well as detected in 19-day-old seedlings (Figure 3C) and 1-month-old seedlings (Figure 3D). In addition, GUS signaling was also detected in stigma, stamen, and siliques (Figure 3E,F). When a 10-day-old p*PtSAP13*::*GUS* transgenic plant was treated with salt for 12 h, strong GUS activity was detected (Figure 3H), which confirms the results of the expression profile of *PtSAP13*.

### 2.4. Effect of Salt Stress on Seed Germination in Transgenic PtSAP13 Arabidopsis Lines

**Figure 4:**
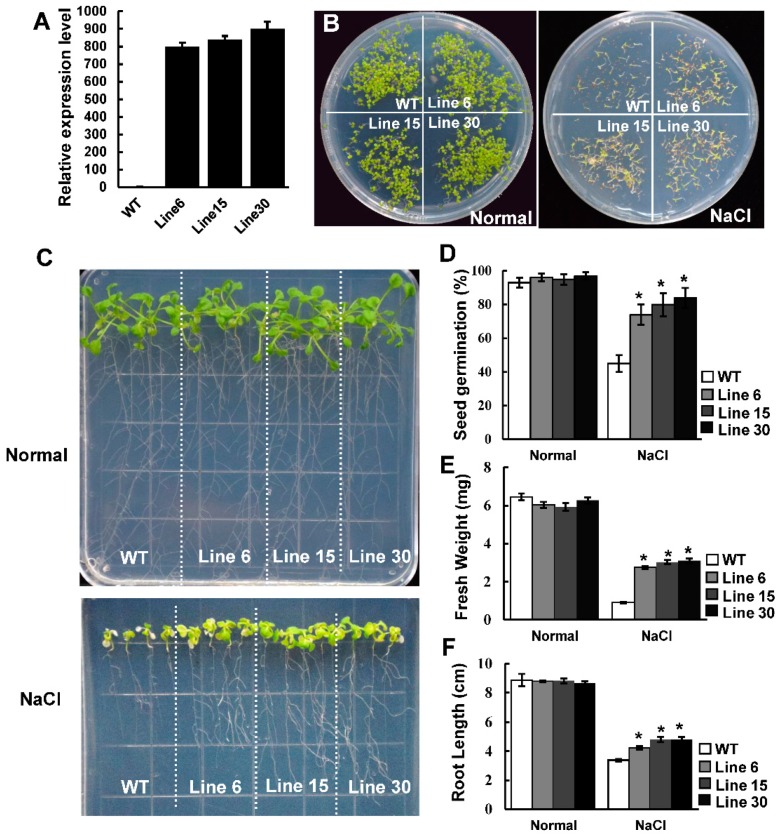
Salt tolerance analysis in panel growth condition. (**A**) qRT-PCR analysis of *PtSAP13* expression in wild-type (WT) and transgenic plants. (**B**) Seed germination of WT and transgenic lines under normal and salt medium. (**C**) Photograph of WT and transgenic seedlings under normal and salt treatment medium. (**D**–**F**) Measurement of the seed germination (**D**), fresh weight (**E**), and root length (**F**) of WT and *PtSAP13*-overexpressing plants under normal condition and stress treatments. Error bars indicate the standard deviations, and * indicate significant differences compared with WT at the *p* < 0.05 level.

To gain insight into the function of *PtSAP13* in salt stress tolerance, transgenic *Arabidopsis* lines with overexpressing *PtSAP13* were generated. Three transgenic lines (Line 6, Line 15, and Line 30) with high expression of *PtSAP13* were used for its stress tolerance investigation (Figure 4A). On the normal 1/2 MS medium, there was no difference in germination rates between the WT and *PtSAP13*-overexpressing plants, and the germination rate was nearly 100% (Figure 4E). After being cultured on 200 mM NaCl medium for 7 days, the germination rates of the three transgenic lines (Line 6, Line 15, and Line 30) were 74.5%, 80.7%, and 84.0%, respectively; whereas the WT exhibited a lower germination rate, with nearly 45.4%, than transgenic lines (Figure 4D). This result indicates that overexpression of *PtSAP13* increased the seed germination under salt stress.

### 2.5. PtSAP13 Transgenic Arabidopsis Seedling Resistance to Salt Stress

**Figure 5:**
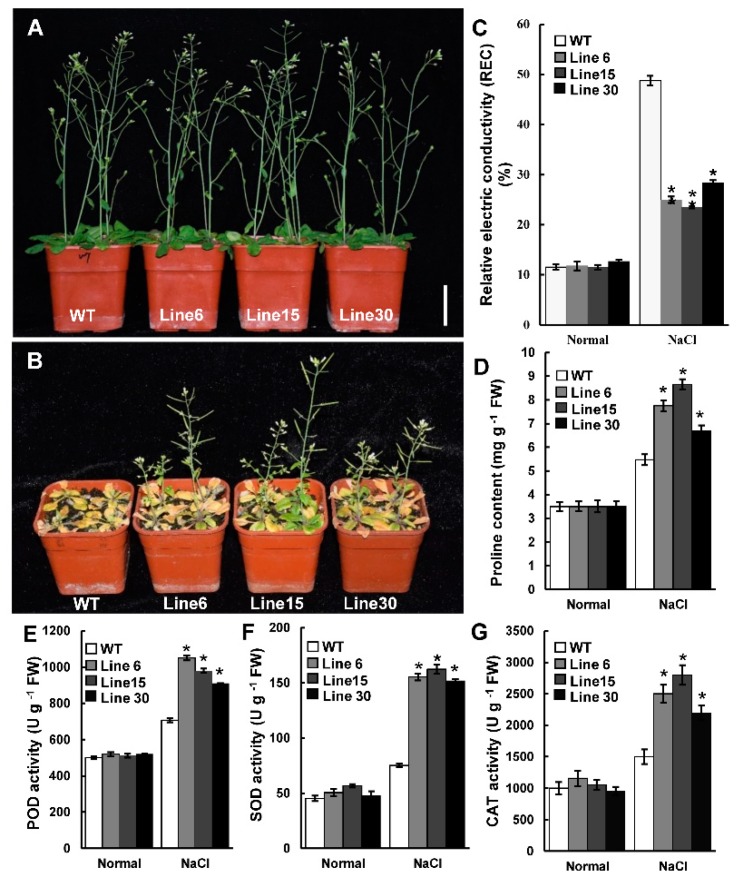
Salt stress tolerance analysis in soil growth condition. (**A**,**B**) Photograph of WT and transgenic seedlings under normal condition (**A**) and salt treatment (**B**). (**C**–**G**) Measurement of the Relative electric conductivity (REC) (**C**), proline content (**D**), POD activity (**E**), SOD activity (**F**), and CAT activity (**G**). Error bars indicate the standard deviations, and * indicates significant differences compared with WT at the *p* < 0.05 level.

One-week-old WT and *PtSAP13*-overexpressing plants were transferred to 1/2 MS solid medium containing 0 mM (normal) or 150 mM NaCl (salt stress). No significant phenotypic difference was observed between the WT and transgenic plants under normal medium (Figure 4C). Under salt condition, the growth of the WT and transgenic plants was significantly inhibited, but the growth situation of the transgenic plants was better than that of the WT plants (Figure 4C). Both average fresh weight and root length of the transgenic plants were more than the WT plants (Figure 4E,F). Furthermore, similar results were also obtained in the soil-grown WT and transgenic plants (Figure 5A,B). The relative electric conductivity in the transgenic plants was lower than in the WT plants (Figure 5C), whereas the proline content was higher in the transgenic plants than in the WT plants under salt condition (Figure 5D). The peroxidase (POD), Superoxide dismutase (SOD), and catalase (CAT) activities of the transgenic plants were higher than that of the WT plants after salt treatment (Figure 5E–G), indicating that scavenging of reactive oxygen species (ROS) was enhanced by *PtSAP13* in transgenic plants. All of these results indicate that overexpression of the *PtSAP13* improved salt tolerance.

### 2.6. Overexpression of PtSAP13 Induced the Expression of Stress-Related Genes

**Figure 6:**
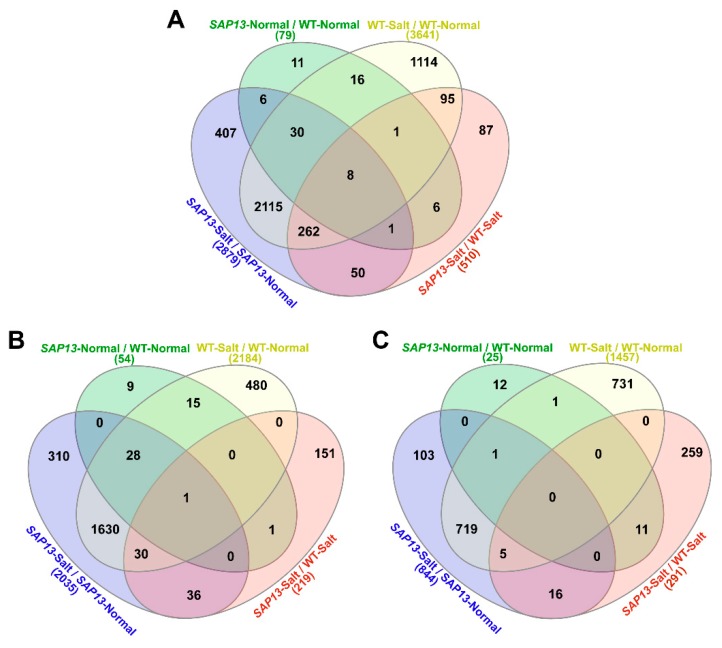
Venn diagram of the differentially expressed genes (DEGs) in four types of comparisons in WT and *PtSAP13*-overexpressing plants under control (normal) and NaCl (salt) conditions. (**A**) Overlapping of DEGs. (**B**) Overlapping of up-regulated genes. (**C**) Overlapping of down-regulated genes. The numbers in brackets represent the total numbers of differentially expressed genes in different comparisons.

To further investigate the regulatory mechanism of *PtSAP13*, RNA-seq analysis was performed for the WT and *PtSAP13*-overexpressing plants under normal and salt conditions. A total of 79 differentially expressed genes (DEGs), including 54 up-regulated genes and 25 down-regulated genes, were identified in the *PtSAP13*-overexpressing plants compared to the WT plants under normal condition (*SAP13*-Normal/WT-Normal) (Figure 6). Compared with the normal condition, 3641 DEGs (2184 up-regulated genes and 1457 down-regulated genes) were detected in WT plants under salt treatment (WT-Salt/WT-Normal), and 2879 DEGs (2035 up-regulated genes and 844 down-regulated genes) in the *PtSAP13*-overexpressing plants under salt treatment (*SAP13*-Salt/*SAP13*-Normal) (Figure 6). In addition, 510 DEGs (219 up-regulated genes and 291 down-regulated genes) were found in the *PtSAP13*-overexpressing plants compared to the WT plants under salt treatment (WT-Salt/*SAP13*-Salt) (Figure 6).

Comparative analysis revealed that 2145 DEGs were overlapped between *SAP13*-Salt/*SAP13*-Normal and WT-Salt/WT-Normal (Figure 6). Notably, 464 DEGs were specific in the *PtSAP13*-overexpressing plants under salt stress (*PtSAP13*-Salt/*PtSAP13*-Normal) but not in the WT plants under salt stress (WT-Salt/WT-Normal) (Figure 6). Among these DEGs, some stress-related genes, including *BZIP60*, *DI19-4*, *Glutathione peroxidase* (*GPX8)*, *NADP-malic enzyme 2* (*NADP-ME2*), *Response to ABA and Salt 1 (RAS1*), *WRKYs* (-*14*, *-24*, -*35*, and -*65*), *Glutathione S-Transferase* (*GSTUs* -*20* and *GSTU22*), and *MYBs* (-*29*, -*67*, -*69*, -*76*, and -*93*), were up-regulated in the transgenic plants under salt condition.

**Figure 7:**
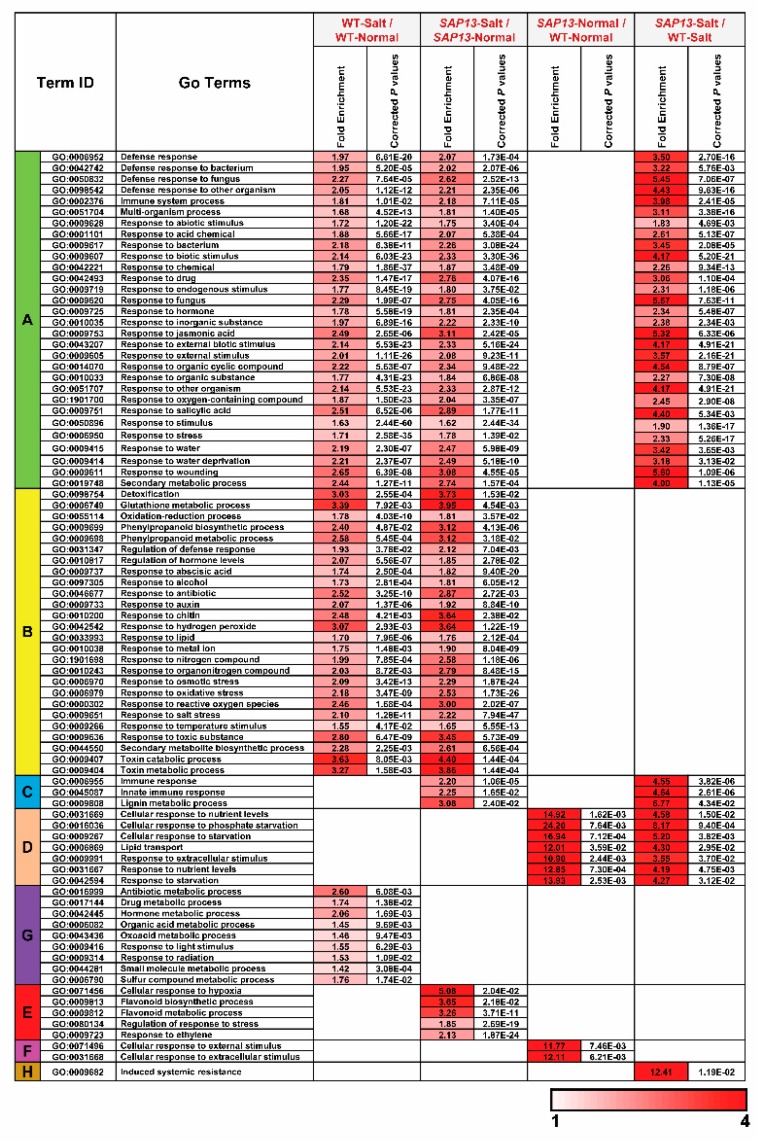
Biological processes in gene ontology (GO) enrichment analysis of all DEGs in four types of comparisons in WT and *PtSAP13*-overexpressing plants. Only significantly enriched terms with corrected *p*-value < 0.05 are indicated.

**Figure 8:**
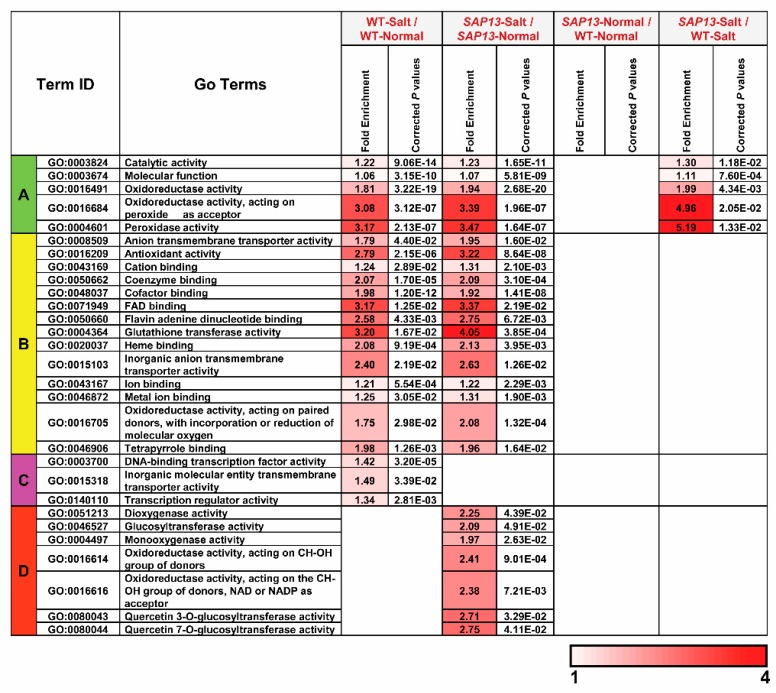
Molecular function in GO enrichment analysis of all DEGs in four types of comparisons of WT and *PtSAP13*-overexpressing plants. Only significantly enriched terms with corrected *p*-value < 0.05 are indicated.

To gain insight into the major functional categories represented by the DEGs, GO enrichment analysis was performed in four comparison sets (WT-Salt/WT-Normal, *SAP13*-Normal/WT-Normal, *SAP13*-Salt/*SAP13*-Normal, and *SAP13*-Salt/WT-Salt). The results show that DEGs were enriched in 83 BP GO terms (Figure 7). A considerable proportion of the GO terms, including multiple responses to abiotic or biotic stresses, responses to various hormones, glutathione metabolic process, oxidation–reduction process, phenylpropanoid biosynthesis and metabolic process, and toxin catabolic and metabolic process, were mainly enriched in WT-Salt/WT-Normal and *SAP13*-Salt/*SAP13*-Normal, implying that these pathways were universal and common response mechanisms under salt stress in both the WT and *PtSAP13*-overexpressing plants (Figure 7). The GO terms of flavonoid biosynthesis and metabolic process, regulation of response to stress, response to ethylene, and cellular response to hypoxia were uniquely enriched only in *SAP13*-Salt/*SAP13*-Normal. In addition, DEGs were enriched in 29 MF GO terms (Figure 7). The dioxygenase activity, glucosyltransferase activity, monooxygenase activity, and oxidoreductase activities acting on CH–OH group of donors and NAD or NADP as acceptor were uniquely enriched only in *SAP13*-Salt/*SAP13*-Normal (Figure 8). These results suggested that *PtSAP13*-overexpressing plants might possess specific regulatory pathways to enhance their salt tolerance (Figure 8).

## 3. Discussion

The number of *SAP* genes increased from lower algae (two *SAPs* in *V. carteri*) through early land plants (from three *SAPs* in *M. polymorpha* to six *SAPs* in *S. moellendorffii*), to higher plants (from 11 *SAPs* in *O. sativa* to 19 *SAPs* in *P. trichocarpa*). *P. trichocarpa* had the maximum number of *SAPs*, suggesting that *PtSAP* genes underwent expansion through whole genome duplication. Previous studies have found that the *HTK1* (*high affinity K^+^ transporter 1*) family in *P. euphratica* [37] and *Hsfs* (*Heat shock transcription factors*) family in *P. trichocarpa* [38] are also expanded to defense against abiotic stresses. *P. trichocarpa* is widely distributed across western North America, with wide geographical and environmental gradients [39]. In addition, compared with herbaceous plants, *P. trichocarpa* has a long life span, thus it might have evolved sophisticated adaptive systems that need the participation of more stress-related genes to ensure it survives in prolonged and repeated environmental stresses.

The expression patterns of *PtSAPs* under salt stress were analyzed. We found that most of *PtSAPs* were induced under salt stress, and similar results have been found in *SAPs* from other species. For example, *Gossypium hirsutum GhSAPs* (*-7A, -7D*, *-8A*, *-11D*, and *-16D*) and *P. euphratica PeuSAPs* (*-1*, *-2*, *-8/14*, and *-16/17*) were significantly induced under salt treatment [15]. Among these *PtSAPs*, *PtSAP13* exhibited more significant up-regulation than other *PtSAPs* under salt stress, suggesting that *PtSAP13* might be involved in salt tolerance in *P. trichocarpa*. Structural analysis found that PtSAP13 protein contained two AN1 domains without AN20. The specific role of AN1 domain is still poorly known. Two SAP proteins, including *Arabidopsis* AtSAP13 and *T. aestivum* TaSAP17-D, that contain two AN1 domains have been reported to enhance the salt tolerance in transgenic plants [33]. In our study, overexpression of *PtSAP13* also improved salt tolerance of transgenic plants, as evidenced by the better growth in fresh weight and root length, as well as increased activities of antioxidative enzymes than WT plants under salt treatment. These results further demonstrate that SAPs containing AN1–AN1 had conserved function and played essential roles in salt tolerance.

Salt stress can trigger two types of stresses, including osmotic stress and ion toxicity. Early effects of salt stress are to prevent plants from absorbing water, and quickly cause decrease of growth rate [40]. During this process, many physiological and transcriptional responses are identical to those caused by water stress [13,40,41]. After excessive amounts of Na^+^ and Cl^−^ enter the plants, ion toxicity will eventually give rise to premature senescence of leaves and limit plant growth and development [13]. To adapt to salt stress, plant have evolved a complex regulatory network that mediates multiple genes [13,41]. In our study, numerous DEGs (2879 DEGs in *PtSAP13*-overexpressing plants and 3641 DEGs in WT plants) were detected after salt treatment, and these DEGs were enriched in multiple biological pathways. These results confirm that plant response and tolerance to salt stress is a complex system. Transcriptome data revealed that some stress-related genes, such as *BZIP60, DI19*-*4*, *GPX8*, *NADP-ME2*, *RAS1*, *WRKYs*, *GSTUs,* and *MYBs*, were up-regulated in the *PtSAP13*-overexpressing plants, but did not change in the WT plants under salt stress. *BZIP60*, a member of basic leucine zipper transcription factor, is involved in plant immunity and abiotic stress responses [42]. Overexpression of *TabZIP60* from *T. aestivum* can enhance the resistance of transgenic *Arabidopsis* to abiotic stresses and enhance ABA sensitivity [43]. *DI19*-*4* is a member of the *Drought-Induced* gene family, its expression in *Arabidopsis* is induced by salt and dehydration [44,45]. Overexpression of *OsDI19*-*4* from *O. sativa* increases stress tolerance by enhancing ROS-scavenging activity [44,45]. *GPX8*participates to withstand oxidative damage and/or to modulate oxidative signaling caused by reactive oxygen species. In the mutant of *gpx8*, the plant exhibits increased sensitivity to oxidative damage by enhancing the oxidized proteins, while overexpression of *Arabidopsis AtGPX8* increases the oxidative tolerance [46]. The expression of *O. sativa NADP-ME2* is induced by NaHCO_3_, NaCl, and PEG stresses. Overexpression from *O. sativa* and *SbNADP-ME* from *Sorghum bicolor* enhances tolerance of transgenic plants’ salt stress [47,48]. *RAS1* is associated with salt tolerance and ABA sensitivity using QTL mapping in *Arabidopsis* [49]. Although there are no reports of *WRKYs* (-*14*, *-24*, -*35*, and *-65*) functions in salt tolerance, multiple members of the *WRKY* family, including *Arabidopsis AtWRKYs* (-*11*, -*17*, -*20*, and -*25*) and *G. hirsutum GhWRKYs* (-*17*, -*34*, -*39*-*1*, and -*41*), have been confirmed to enhance plant salt tolerance [50,51,52,53,54,55]. The expression levels of these genes increased in the *PtSAP13*-overexpressing plants under salt stress, suggesting that *PtSAP13* might enhance plant salt tolerance by collaborating with these stress-related genes.

Salt stress causes excessive generation of ROS, which gives rise to oxidative damage to DNA, proteins, and lipids [56]. To cope with the oxidative damage, plants activate defense systems mediated by enzymatic and non-enzymatic antioxidants to maintain ROS homeostasis [56]. Enzymatic antioxidants, such as POD, SOD, and CAT, play important roles in scavenging ROS and maintaining redox equilibrium [57]. In our study, the activities of these enzymatic antioxidants were higher in *PtSAP13*-overexpressing plants than in the WT plants under salt stress, indicating that the ability of scavenging ROS was enhanced by *PtSAP13* in transgenic plants. Flavonoids have been identified as non-enzymatic antioxidants to scavenge ROS and protect plants from oxidative damage [58]. Overexpression of *Vitis amurensis NAC26* and *Antirrhinum AmDEL* significantly increases the accumulation of flavonoids and enhances salt tolerance in transgenic plants [59,60]. In *PtSAP13*-overexpressing plants, flavonoid biosynthesis and metabolic processes were uniquely enriched under salt stress, which might help mediate the salt tolerance of transgenic plants.

In addition, water and nutrients were transported in conductive tissues to maintain plant growth. When plants are subjected to salt stress, their nutrient homeostasis is disrupted [61]. Improvement of the ability to absorb nutrients and increase amounts of essential nutrients will help plants resist the salt stress [61,62]. In our study, *PtSAP13* was strongly expressed in the conductive tissues (Figure 3), and its expression was induced under nitrate and urea condition (Figure S2). Thus, we speculated that *PtSAP13* might also be involved in uptake and/or transport of nutrients. We also found that the nutrient-related biological processes, including the pathways of celluar response to nutrient levels and response to starvation, were enriched in *SAP13*-Normal/WT-Normal and *SAP13*-Salt/WT-Salt. Thus, these results further indicate that *PtSAP13* might enhance salt tolerance by regulating nutrient-related processes.

## 4. Materials and Methods

### 4.1. Plant Materials and Salt Treatments

Seeds of *A. thaliana* (ecotype Columbia) which conserved in Chinese Academy of Forestry were surface-sterilized with 75% (v/v) ethanol twice, followed by 100% (v/v) ethanol once in clean bench. After being dried on filter paper, the seeds were sowed on 1/2 Murashige-Skoog (MS) solid medium containing 0.8% agar. After being vernalized at 4 °C for 3 days in the dark, the seeds were grown at 20–22 °C under long-day conditions (16/8 h light/dark) in medium or soil.

Two-month-old clones of *P. trichocarpa* were water-cultured using Hoagland solution with 150 mM NaCl for salt treatment. Fully matured leaves from six clones were immediately harvested after 0, 1, 6, 12, and 48 h of salt treatment and stored at −80 °C for further expression analysis. The dosages of the salt treatment were determined based on previous study [63]. This experiment included three biological replicates.

### 4.2. Gene Expression Analysis of PtSAPs

Expression data of *PtSAPs* were obtained from *Populus* Gene Atlas database (https://phytozome.jgi.doe.gov/pz/portal.html), including leaf: Leaf immature standard (1), leaf young standard (2), and leaf first fully expanded standard (3); root: Root standard (4), root tip standard (5), root ammonia (6), root nitrate (7), and root urea (8); stem: Stem node standard (9), stem inode standard (10), stem ammonia (11), stem nitrate (12), and stem urea (13); bud-related: Pre-dormant bud I (14), pre-dormant bud II (15), early dormant bud (16), late dormant bud (17), and fully open bud (18); male catkin-related: Male early GW9592.ZK 10 (19), GW9840.ZE 30 (20), and male mid GW9911.ZK 51 (21); female catkin-related: Female early BESC423.ZL 7 (22), female late BESC842.ZI 22 (23), and female receptive BESC443.ZG 43 (24). The expression levels of *PtSAPs* across the tissues, developmental processes, or treatments were compared with the mid-value. The fold changes were log_2_ transformed for the heatmap.

### 4.3. RNA Isolation and Quantitative Real-Time PCR (qRT-PCR) Analysis

An RNeasy Plant Mini Kit (Qiagen, Hilden, Germany) was used for extracting RNA from plant samples, according to the manufacturer’s instructions. During the extraction process, DNase I (Qiagen, Hilden, Germany) was used to degrade genomic DNA. The SuperScript III first-strand synthesis system (Life technologies, Carlsbad, CA, USA) was used to synthesize the first-strand cDNA with 3 μg mRNA. The qRT-PCR was performed on the Roche LightCycler 480 (Roche Applied Science, Penzberg, Germany) using SYBR Premix Ex Taq (Takara, Kusatsu, Japan). Each sample was performed in three biological replicates and four technical replicates. *AtActin2* (*AT3g18780*) and *PtActin* (*Potri.001G309500*) were used as reference genes in *Arabidopsis* and *P. trichocarpa*, respectively. The 2^−ΔΔCT^ method was used to calculate the relative expression levels of each gene [64]. The primers used for qRT-PCR in this study are listed in Table S2.

### 4.4. Generation of Transgenic A. thaliana Plants

The coding sequence of *PtrSAP13* was amplified from the cDNA of *P. trichocarpa* using gene-specific primers (Table S2). The amplified fragment was cloned into the pDONR222 vector (Life technologies, USA) to produce pENTR for sequencing. The correct sequence was sub-cloned into pMDC32 to construct the plasmid p*35S*::*PtSAP13*, which was used to generate overexpressing transgenic plants. The promoter sequence of *PtSAP13* was amplified from the genomic DNA of *P. trichocarpa* and cloned into the pMDC164 vector to construct the plasmid p*PtSAP13*::*GUS,* which was used to study the expression pattern of *PtSAP13.* The correct constructs described above were transformed into *Agrobacterium GV3101* by electroporation and transform *A. thaliana* using the floral dip method [65]. The positive transgenic plants were selected on 1/2 MS plates containing 25 mg/L hygromycin and PCR identification. More than 30 independent transgenic lines of p*PtSAP13*::*GUS* and 31 independent transgenic lines of p*35S*::*PtSAP13* were obtained, respectively. Finally, three transgenic lines of p*PtSAP13*::*GUS* were used for GUS staining, according to previous research [66]. Three transgenic lines with high abundance of *PtSAP13* were used for further salt tolerance analysis and RNA-Seq analysis.

### 4.5. Analysis of Salt Tolerance

For the seed germination assays, T3 homozygous seeds (100–150 seeds) of transgenic lines and wild-type (WT) were sown on 1/2MS medium containing 0 mM (normal) or 200 mM NaCl (salt stress). After being vernalized at 4 °C for 3 days in the dark, seeds were transferred under normal conditions, and seed germination was counted after 1 week. Germination was scored as rupture of the seed coat.

For the salt tolerance experiments of seedlings, 1-week-old *PtSAP13*-overexpressing and WT plants were cultured on 1/2 MS medium containing 0 mM or 150 mM NaCl for 2 weeks, and the fresh weight and root length were measured. In addition, after growth on 1/2 MS normal medium for 2 weeks, the transgenic and WT plants were transferred on soil for 4 weeks, and then irrigated with 30 mL of 200 mM NaCl for 2 weeks, and followed with pure water for 7 days. The relative electric conductivity, proline content, and enzyme activities were measured after salt tolerance experiments. The relative electric conductivity was determined by conductivity meter (DDS-307). The proline content was analyzed according to Dubois et al. [67]. The activities of SOD, POD, and CAT were measured according to Li et al. [63]. All experiments were performed in three biological replicates. The data were analyzed with a *t*-test to detect significant differences.

### 4.6. RNA-Seq Analysis of Transgenic Arabidopsis

The seeds from WT and transgenic plants were grown under normal condition for 1 week. Subsequently, the seedlings were transferred into 1/2 MS medium containing 0 mM or 150 mM NaCl for 2 weeks. Finally, the whole plants of WT under normal and salt conditions were collected and marked as WT-Normal and WT-Salt, respectively; three independent transgenic lines under normal and salt conditions were equally pooled and marked as *SAP13*-Normal and *SAP13*-Salt, respectively.

Total RNA was also extracted using RNeasy Plant Mini Kit (Qiagen, Hilden, Germany) and treated with DNase I (Qiagen, Hilden, Germany). The RNA purity and integrity were detected by the NanoPhotometer spectrophotometer and Agilent2100 Bioanalyzer, and the RNA quantity was measured by Qubit2.0 Fluorometer (Life Technologies, New York, USA). The cDNA libraries were constructed and sequenced on the Illumina HiSeq2500 sequencing platform by Gene Denovo Biotechnology Co. (Guangzhou, China), with paired-end sequencing and read lengths of 150 bp. Each sample was performed for three biological replicates. Quality-control procedure was performed to filter adapter sequences and low-quality reads using fastp (version 0.18.0) (https://github.com/OpenGene/fastp) [68]. The clean data were obtained for subsequent analyses. Clean reads were mapped to the reference genome using HISAT2 (version 2.4) (http://ccb.jhu.edu/software/hisat/index.shtml) [69]. Gene expression levels were calculated as reads per kilobase of transcript sequence per million base pairs sequenced (FPKM) using StringTie (version 1.3.1) (https://ccb.jhu.edu/software/stringtie/)) [70]. The differentially expressed genes (DEGs) were identified by DESeq2 (https://bioconductor.org/packages/release/bioc/html/DESeq2.html) [71]. Genes with |log_2_FoldChange| >1.0 and false discover rate (FDR) <0.05 were considered differentially expressed. To study the biological function of DEGs, gene set enrichment with gene ontology (GO) terms was performed using Blast2GO (version 5.2.5) (https://www.blast2go.com/) [72]. Fischer’s exact test was used to assess the significance of GO categories*. p-*values were corrected by Benjamini–Hochberg FDR and the corrected *p*-value cut-off was 0.05. RNA-Seq data have been deposited in the NCBI Sequence Read Archive under accession number PRJNA579744.

## 5. Conclusions

In our study, we isolated *PtSAP13* from *P. trichocarpa* and characterized its function in salt tolerance. *PtSAP13* was highly induced by salt stress and constitutive expression of *PtSAP13* increased the tolerance to salt stress. The *PtSAP13*-overexpressing plants enhanced salt tolerance by improving ROS-scavenging ability. Moreover, RNA-seq data showed that some stress-related genes were significantly induced in *PtSAP13*-overexpressing plants, suggesting that *PtSAP13* may paly a role in plant salt tolerance by regulating the expression of these genes, although detailed work will be needed to identify the exact regulatory mechanism of *PtSAP13* in stress tolerance in poplar.

## Supplementary Materials

Supplementary materials can be found at https://www.mdpi.com/1422-0067/20/22/5782/s1. Figure S1: Multiple sequence alignment of the PtSAP proteins. The A20 domain, AN1 domain, and CH2–CH2 domain are marked with red, black, and rule boxes, respectively. Figure S2: The expression patterns of *PtSAP* genes across different tissues. (1) Leaf immature standard, (2) leaf young standard, (3) leaf first fully expanded standard, (4) root standard, (5) root tip standard, (6) root ammonia, (7) root nitrate, (8) root urea, (9) stem node standard, (10) stem inode standard, (11) stem ammonia, (12) stem nitrate, (13) stem urea, (14) pre-dormant bud I, (15) pre-dormant bud II, (16) early dormant bud, (17) late dormant bud, (18) fully open bud, (19) GW9592.ZK 10 male early, (20) GW9840.ZE 30 male early, (21) GW9911.ZK 51 male mid, (22) BESC423.ZL 7 female early, (23) BESC842.ZI 22 female late, (24) BESC443.ZG 43 female. All the expression data was log_2_ transformed. Table S1: The detailed *SAP* gene information and protein sequences in different species. Table S2: The primers sequence used in the study.
