# Supplementary figures and images for: A Stress-Associated Protein, PtSAP13, From Populus trichocarpa Provides Tolerance to Salt Stress
Source: Int J Mol Sci. 2019 Nov 17;20(22):5782. doi: 10.3390/ijms20225782 (PMC6888306; doi:10.3390/ijms20225782)

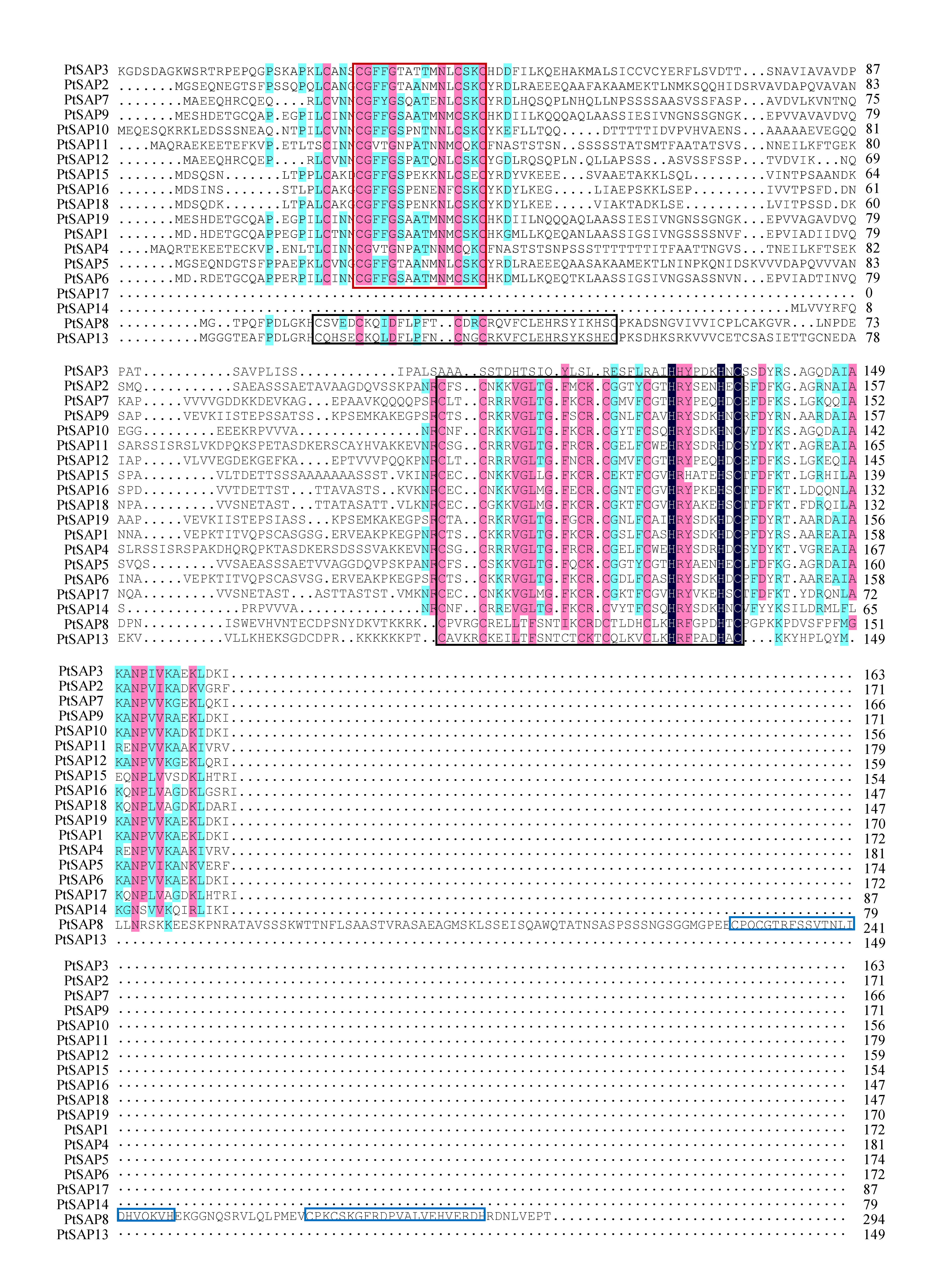

Supplement: Supplementary file 1 [file ijms-20-05782-s001.zip › Supplementary files/Figure S1.tif]
